# A Novel Ultrafiltration Rate Feedback Controller for Use in Hemodialysis: First Clinical Experience: An Interventional Pilot Study

**DOI:** 10.34067/KID.0000000839

**Published:** 2025-06-04

**Authors:** Stephan Thijssen, Lemuel Rivera Fuentes, Leticia Mirell Tapia Silva, Xiaoling Ye, Sabrina Casper, Doris H. Fuertinger, Stefan Fuertinger, Peter Kotanko

**Affiliations:** 1 Renal Research Institute, New York, New York; 2 Fresenius Medical Care Deutschland GmbH, Bad Homburg, Germany; 3 Icahn School of Medicine at Mount Sinai, New York, New York

**Keywords:** hemodialysis, ultrafiltration

## Abstract

**Key Points:**

- The ultrafiltration rate feedback controller functioned as intended, improving relative blood volume target attainment over standard care.
- Predialytic, postdialytic, and mean intradialytic BPs were not statistically different between treatments with versus without controller usage.
- Intradialytic nadir BP was on average slightly lower with use of the controller (106 versus 111 mm Hg systolic).

**Background:**

Relative blood volume (RBV) monitors are increasingly being used during hemodialysis. Manual ultrafiltration rate (UFR) adjustments to establish a favorable RBV trajectory are not feasible in routine practice. The goal of this study was to characterize the behavior of a new UFR feedback controller *in vivo*.

**Methods:**

In this pilot trial, chronic hemodialysis patients were prospectively studied during up to six successful study dialysis treatments each. During each study visit, the feedback controller generated UFR recommendations designed to guide the subject's RBV curve toward a predefined target trajectory. Each recommendation was evaluated by licensed health care staff and then either implemented or disregarded. The results were compared with standard-of-care treatments in the same subjects.

**Results:**

Fifteen subjects (age 59±15 years, eight men) were studied during a total of 63 treatments. The controller functioned as intended and issued a total of 1037 recommendations. Compared with standard-of-care treatments, its use was associated with a higher probability of RBV target range attainment (69% versus 47%) and lower nadir systolic (106 versus 111 mm Hg) and diastolic (55 versus 59 mm Hg) BP.

**Conclusions:**

The UFR feedback controller operated as intended, and its use led to a substantial increase in the rate of RBV target range attainment. This technology holds promise for improving fluid management in chronic hemodialysis patients.

## Visual Abstract

**Figure:**
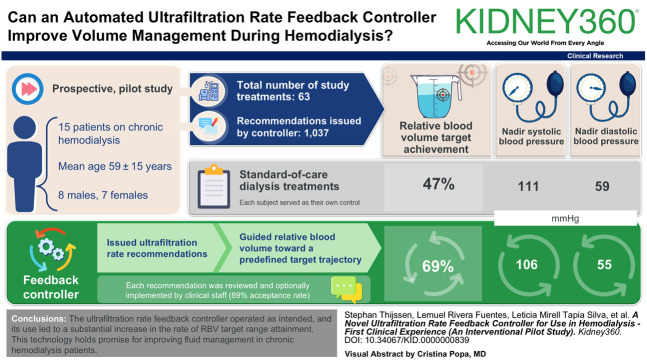

## Introduction

Fluid management is a major challenge for the approximately 500,000 patients with ESKD in the United States undergoing hemodialysis.^1–4^ Vascular refilling varies between and within patients,^5–7^ making it difficult to determine the tolerable fluid removal amount per treatment. Relative blood volume (RBV) monitors, which provide real-time insights into the intravascular compartment during the treatment, are increasingly used. However, ideal RBV targets have historically been ill-defined. In addition, the varying vascular refilling rate during the treatment would require constant ultrafiltration rate (UFR) adjustments by the nurse to meet RBV targets, which is not practical.

Preciado *et al.* addressed the first challenge by publishing half-hourly RBV target ranges associated with improved patient survival.^8^ To tackle the second challenge, a closed-loop control algorithm was developed to provide UFR recommendations that guide a patient's RBV curve toward a prespecified target trajectory based on Preciado's data.^9^ This algorithm, referred to as “UFR Feedback Controller” or “Controller” in this article, is the first of its kind that targets an RBV trajectory associated with improved patient survival.

In this pilot study, we are characterizing this novel adaptive UFR Feedback Controller's behavior. The primary aim was to technically validate the Controller's operation as a prerequisite for larger clinical trials. Although clinical outcomes were not the main focus of this study, we provide some preliminary observations that may inform the design of future trials.

## Methods

### Study Aims

The primary aim of this pilot study was to establish whether this novel UFR Feedback Controller technically operates as intended, as assessed by (*1*) its abidance by the software-imposed bounds that constrain its operation and (*2*) the appropriateness of any Skipped Updates, Fallback Conditions, and Hard Stops, which are Controller states triggered by certain conditions, as previously described.^9^

Secondary aims were to characterize this novel UFR Feedback Controller with respect to RBV target range attainment, BP, intradialytic morbid events, and health care staff acceptance of the Controller recommendations.

### UFR Feedback Controller

The UFR Feedback Controller's mode of operation has been described previously.^9^ In brief, the Controller is a proportional-integral controller that, in its prototype implementation, runs on a laptop and receives data from the dialysis machine and the Crit-Line (Fresenius Medical Care North America, Waltham, MA) to compute UFR adjustments every 10 minutes (starting at minute 15) based on the patient's RBV curve. These time points are referred to as “Controller update time points.” The ideal RBV trajectory that the Controller aims for is given as the straight-line interpolation between the RBV values that represent the all-cause mortality hazard ratio nadirs for the half-hourly RBV target ranges published by Preciado *et al.*^8^ (from 30 to 180 minutes; Supplemental Table 1), followed by a horizontal RBV trajectory until the end of the treatment. These are considered *RBV target ranges* for the analyses in this study. The Controller communicated its UFR recommendations through a graphical user interface for the health care staff to evaluate and either implement or disregard. The initial Controller prescription requires the clinician to specify a prescribed ultrafiltration (UF) volume and a maximum allowed upward and downward deviation from this goal. All recommendations the Controller makes are always compatible with this initial prescription.

### Study Design

This was a multicenter, single-arm, prospective, interventional pilot study conducted in three Manhattan outpatient dialysis centers from March to September of 2019. The study was reviewed and approved by Western Institutional Review Board-Copernicus Group Institutional Review Board (tracking number: 20183205) and conducted in adherence with the Declaration of Helsinki. Informed consent was obtained from all subjects. Adult patients (18 years or older) were studied during up to six successful study visits. All patients had been on chronic hemodialysis for at least 6 months and had three dialysis sessions per week with a duration of at least 180 minutes each. Exclusion criteria were prescription of intradialytic sodium profiling, scheduled renal transplantation during the study, and simultaneous participation in another clinical study with potential effect on cardiovascular or hemodynamic parameters. Subjects were dialyzed using 2008T/2008T BlueStar hemodialysis machines (Fresenius Medical Care North America, Concord, CA) and high-flux polysulfone dialyzers.

Controller recommendations were evaluated by licensed health care staff. Disregarded Controller recommendations were classified according to the underlying reasons into four categories: (*1*) low BP without clinical symptoms, (*2*) low BP with clinical symptoms, (*3*) clinical symptoms without low BP, and (*4*) health care staff preference in the absence of clinical symptoms or low BP. For this classification, the definition of low BP was left to the clinicians, so as to allow them to register a low BP concern without having to meet a formal threshold. Age, sex, BP (measured every 10 minutes), clinical symptoms, saline administrations, and UF rate/goal changes were recorded. A clinical research coordinator or research scientist was with the study subjects throughout the entire study treatment, and subjects were instructed to immediately report any clinical symptoms they experienced, without restriction. All reported clinical symptoms were documented and evaluated by licensed health care staff. For comparison with standard-of-care (SOC) treatments, RBV and BP data for the study subjects were retrospectively extracted from the electronic medical records.

Before this study, no clinical data or experience with the use of this Controller were available on which a formal power calculation could have been based.

### Statistical Analyses

Descriptive statistics for continuous and categorical variables are reported. A “complete study visit” was defined as a treatment where the Controller was used for at least 180 minutes.

RBV data were available once per second during study treatments and either once every 10 seconds or once a minute during SOC treatments. For analysis of RBV target range attainment, we averaged RBV values from 10 minutes before to 10 minutes after each target time point in accordance with Preciado *et al*.^8^

For analyses of RBV target range attainment in SOC treatments, we considered complete treatments that were within 90 days before the first study visit or 90 days after the last study visit, from subjects who contributed at least two such treatments to each study period (SOC treatments and study visits, respectively). For BP analyses in SOC treatments, we considered both complete and incomplete treatments that were within 90 days before the first study visit or 90 days after the last study visit, from subjects who contributed at least one such treatment to each study period. For BP analyses, only data from minute 10 to minute 190 were included.

For group comparisons, both parametric and nonparametric paired testing was performed (paired *t* test and Wilcoxon matched-pairs signed rank test). For confirmation of those results, generalized linear mixed models were constructed with study period (study visits versus SOC treatments) as fixed effect and study subject as random effect. Effect size was assessed using Cohen *d*. A *P* value < 0.05 was deemed statistically significant.

Analyses were conducted using R version 4.4.0.^10^ Plots were created with ggplot2. Other packages used: plyr, dplyr, reshape2, tidyverse, knitr, bookdown, kableExtra, and xfun.

## Results

### Study Subjects and Study Visits

Fifteen subjects (age 59±15 years, eight men) were studied during a total of 63 treatments (per-subject median: 4; minimum: 1; first quartile: 3; third quartile: 5.5; maximum: 8 treatments). Fourteen subjects had at least one complete study visit, and eight subjects had at least four complete study visits. We conducted a total of 48 complete study visits (37 from subjects with at least four complete visits). Study population characteristics are presented in Table 1. An exploratory analysis comparing subjects with a high versus low rate of disregarded Controller suggestions showed a trend toward longer dialysis vintage in patients with a higher rate of disregarded Controller suggestions (5.7±2.7 versus 3.0±1.6 years, *P* = 0.063; Supplemental Table 2). No other clinically meaningful differences were observed between the two groups.

**Table 1:** Study population characteristics

Parameter	All Subjects	Subjects with ≥4 Complete Study Visits
No. of subjects	15	8
Age (yr)	59±15	57±19
Sex: female (%)	47	38
Dialysis vintage (yr)	4.1±2.4 (min 0.5; max 9.9)	4.5±3 (min 1.4; max 9.9)
Body mass index (kg/m^2^)	30.4±12.2 (min 18.5; max 67.3)	27±8.2 (min 18.5; max 43.4)
Baseline IDWG (kg)	2.6±0.8	2.5±0.8
Baseline prehemodialysis weight (kg)	87.2±35.4	78.6±24.9
Baseline posthemodialysis weight (kg)	84.4±34.9	75.9±24.5
Baseline clin. target weight (kg)	83.3±35	75.1±24.9
Prescribed treatment time (min)	222±28	217±23

Baseline parameters were defined as the average of the three values from the most recent dialysis sessions after the long, first short, and second short interdialytic interval before the first study visit. Body mass index was calculated using the baseline postdialysis weight. IDWG, interdialytic weight gain.

### Study Visits without Valid Data

In 4 of the 63 conducted study visits, no analyzable data are available because the study visits were ended shortly after starting the treatment (in three cases due to a graphical user interface malfunction and in one case due to air bubbles in the Crit-Line blood chamber).

### Incomplete Study Visits

Of the 59 study visits with analyzable data, 11 visits were incomplete (Supplemental Table 3). In only one case did a medical intervention by the staff lead to the disengagement of the Controller; in UFC-010-3, the UF pump was stopped for approximately 40 minutes due to low BP (of note, at the beginning of the dialysis treatment, before any UF had occurred, the subject already had a BP of only 98/65 mm Hg). When the UF pump was restarted, the remaining UF time exceeded the maximum allowed value for use of the Controller and the Controller appropriately disengaged.

### Technical Controller Performance Metrics

#### Adherence to Imposed Limitations

The UFR Feedback Controller is constrained in its operation by certain bounds.^9^ The following is an analysis of the Controller's abidance by these rules:

- Maximum allowed upward UF goal deviation: respected in 100% of cases.
- Maximum allowed downward UF goal deviation: respected in 100% of cases.
- “175% of prescribed UFR” maximum UFR limitation: respected in 100% of cases.
- 13 ml/kg per hour average UF rate limitation: respected in 100% of cases.
- Relative UF rate increase/decrease limitations: respected in 100% of cases.

#### Appropriateness of Skipped Updates, Fallback Conditions and Hard Stops

In the 59 study visits with analyzable data, the Controller skipped 34 updates, went into Fallback mode eight times, and reached a Hard Stop nine times. All 51 of these conditions were triggered appropriately and handled correctly by the Controller. Seven of the Controller updates were skipped intentionally for testing purposes.

### Attainment of RBV Targets

#### Rates of Attaining Each of the RBV Targets

**Figure 1:**
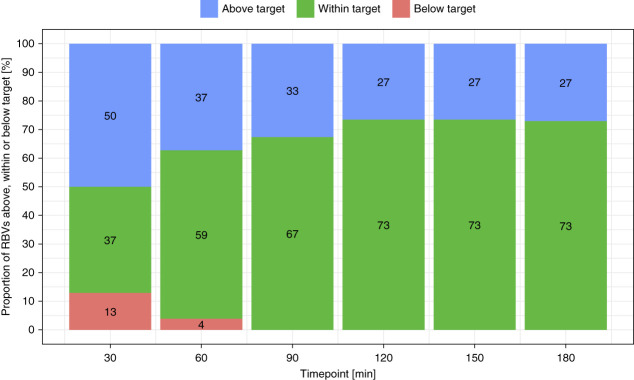
**Proportion of RBV values above, within and below the respective RBV target range for each of the RBV target time points during study treatments with the Controller.** Underlying data: all subjects who contributed data (*n*=14), all RBV targets (300 data points). RBV, relative blood volume.

Fourteen subjects contributed RBV target time points for analysis. Figure 1 shows the proportion of RBV values above, within and below the respective target range for each of the six time points during study treatments with the Controller.

**Figure 2:**
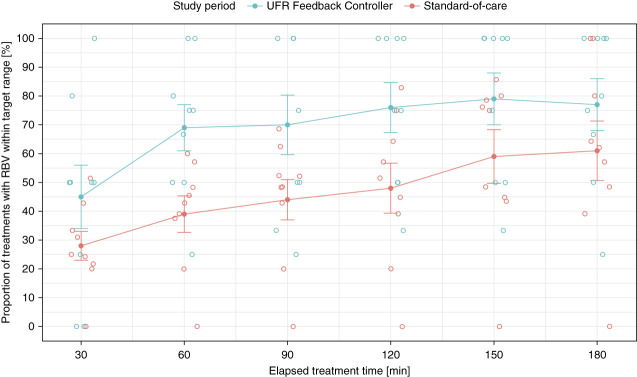
**Proportion of treatments with RBV within the desired target range.** Underlying data: subjects who contributed at least two complete treatments to each study period and whose contributing standard-of-care treatments were within 90 days of the study period (*n*=9 subjects). Open circles denote individual per-subject data, filled circles denote mean across all subjects, and error bars denote SEM. UFR, ultrafiltration rate.

Across all six RBV target time points, the overall probability of achieving an RBV within the desired target range was 47% for SOC treatments versus 69% for study visits using the Controller (*P* = 0.02, parametric; *P* = 0.03, nonparametric; *n*=9 subjects contributing; Cohen *d*=0.96). The probabilities of achieving an RBV within the desired target range for each time point, stratified by study period (SOC versus UFR Feedback Controller), are presented in Figure 2 and Table 2. The use of the Controller was associated with a higher rate of RBV target range attainment for every time point, and these differences were statistically significant for time points 60, 120, and 150 and borderline significant for time points 90 and 180 in paired *t* testing. Nonparametric testing yielded similar results.

**Table 2:** Relative blood volume target attainment by time point

Time Point	Percent of Treatments with RBV in Target: SOC	Percent of Treatments with RBV in Target: UFR Feedback Controller	*P* Value
30	28	45	0.128
60	39	69	0.014
90	44	70	0.065
120	48	76	0.014
150	59	79	0.023
180	61	77	0.066

Underlying data: subjects who contributed at least two complete treatments to each study period and whose contributing standard-of-care treatments were within 90 days of the study period (*n*=9 subjects). *P* values are from paired *t* test. Nonparametric testing yielded similar results. RBV, relative blood volume; SOC, standard-of-care; UFR, ultrafiltration rate.

We confirmed these results with generalized linear mixed-effects models; across all RBV target time points, the use of the Controller was a significant predictor of better RBV target range attainment (*P* < 0.001). When modeled separately for each time point, the modeled probabilities for RBV target range attainment were similar to the descriptive probabilities reported in Table 2. The use of the UFR Feedback Controller was a significant predictor of RBV target range attainment for time points 60, 90, 120, and 150 and a borderline significant predictor for time points 30 and 180, generally in line with the paired *t* test results reported in Table 2.

### BP and Clinical Symptoms

#### BP Differences between Study Visits and SOC Treatments

**Figure 3:**
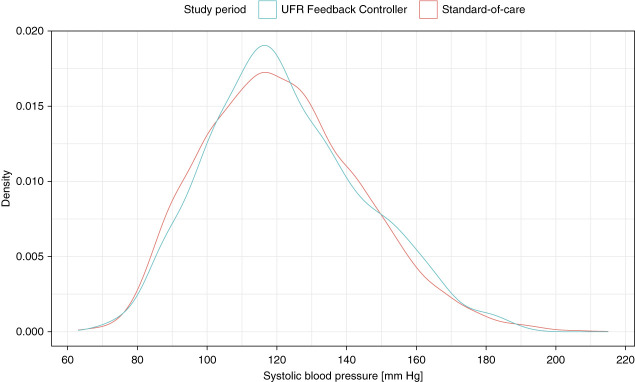
**Density plot of intradialytic SBP, stratified by study period (standard-of-care versus UFR Feedback Controller).** Underlying data: all intradialytic SBP values from subjects who contributed BP measurements to both study periods and whose contributing standard-of-care treatments were within 90 days of the study period (*n*=14 subjects). Both complete and incomplete treatments are included. SBP, systolic BP.

Figure 3 shows the density distribution of systolic BP (SBP) values for SOC treatments and study visits using the UFR Feedback Controller, respectively. Subjects' average systolic and diastolic BPs (DBPs) were not statistically different between SOC treatments and study visits. However, for average nadir systolic and DBP, there was a trend toward lower values during study visits (systolic: 106 versus 111 mm Hg; diastolic: 55 versus 59 mm Hg). For average nadir DBP, the difference reached statistical significance in paired testing. See Table 3 for detailed results. Predialysis SBP was not significantly different between the two study periods (147 mm Hg for SOC; 149 mm Hg for study visits; *P* = 0.6, parametric; *P* = 0.9, nonparametric), and neither was postdialysis SBP (126 mm Hg for SOC; 125 mm Hg for study visits; *P* = 0.7, parametric; *P* = 0.6, nonparametric). The same was true for DBP before (78 mm Hg for SOC; 79 mm Hg for study visits; *P* = 0.4, parametric; *P* = 0.7, nonparametric) and after dialysis (68 mm Hg for SOC; 67 mm Hg for study visits; *P* = 0.4, parametric; *P* = 0.5, nonparametric).

**Table 3:** BP-related metrics

Study Period	Average SBP Across Subjects (mm Hg)	Average DBP Across Subjects (mm Hg)	Average Nadir SBP Across Subjects (mm Hg)	Average Nadir DBP Across Subjects (mm Hg)
SOC	123.4	67.6	110.6	58.7
Study visits (Controller)	123.2	67.2	106.1	55.1
Difference, Controller−SOC (95% confidence limits)	−0.11 (−5.54 to 5.32)	−0.35 (−2.96 to 2.26)	−4.55 (−12.47 to 3.37)	−3.66 (−7.03 to −0.3)
*P* value (paired *t* test)	0.97	0.78	0.24	0.04
*P* value (Wilcoxon)	0.9	0.86	0.07	0.03

All metrics were first averaged on a per-subject level and then further averaged across subjects. Underlying data: subjects who contributed BP measurements to both study periods and whose contributing standard-of-care treatments were within 90 days of the study period (*n*=14 subjects). Both complete and incomplete treatments are included. “Wilcoxon” denotes Wilcoxon matched-pairs signed rank test. DBP, diastolic BP; SBP, systolic BP; SOC, standard-of-care.

When modeled using generalized linear mixed-effects models, the average differences in nadir SBP and nadir DBP, respectively, between SOC treatments and study visits were similar to the ones reported in Table 3, and the use of the Controller was a significant predictor of lower nadir systolic (*P* = 0.01) and diastolic (*P* = 0.004) BP.

#### Clinical Symptoms during Study Visits

The frequencies of intradialytic clinical symptoms observed across all study visits where the Controller was used (59 study visits from 15 subjects) are presented in Table 4. These data were not available for SOC treatments. There was only one instance of a SBP below 90 mm Hg that required a concomitant saline administration (200 ml) during the study.

**Table 4:** Frequencies of clinical symptoms observed during study visits

Type of Event	No. of Events Observed	No. of Study Visits with This Event	Percentage of Study Visits with This Event
Cramping	12	7	12
Dizziness	3	3	5
Chest pain	2	2	3
Diaphoresis	1	1	2
Dyspnea	0	0	0
Nausea	0	0	0
Vomiting	0	0	0

Underlying data: all study visits, complete or incomplete, where the Controller was used (59 visits).

### Controller Suggestions Accepted versus Disregarded

#### Overall Acceptance Rate for Controller Suggestions

Between 56 study visits (48 complete and eight incomplete) from 14 subjects, a total of 1037 UF goal suggestions were made by the Controller. Of these, 926 (89%) were accepted by the health care staff.

#### Treatment-Level Analysis of Implemented versus Disregarded Controller Suggestions

**Figure 4:**
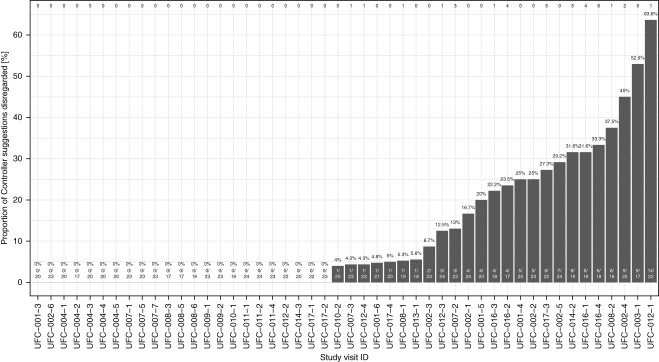
**Proportion of Controller suggestions disregarded by the health care staff for all 48 complete study visits.** The percentage of Controller suggestions disregarded is denoted above each bar. The two numbers along the bottom indicate how many suggestions were disregarded out of how many suggestions in total. The numbers along the top of the chart denote the number of suggestions that were disregarded for acute medical reasons, defined as low BP, clinical symptoms, or both.

For each of the 48 complete study visits, Figure 4 shows the number and proportion of Controller suggestions that were disregarded. In 48% of the study visits, every Controller suggestion was implemented.

For study visits where at least one Controller suggestion was disregarded, in about a quarter of these treatments, the health care staff disregarded only a single Controller suggestion, and in 40% of these treatments, three or fewer suggestions were disregarded. Importantly, not all cases of disregarded Controller suggestions were due to acute medical reasons (defined as low BP, clinical symptoms, or both). The number of suggestions disregarded for acute medical reasons is denoted by the numbers along the top of Figure 4. In those treatments where only one suggestion was disregarded, more than half of the cases were not related to low BP or clinical symptoms. Across all 48 complete study visits, approximately 69% of the treatments did not have a single Controller suggestion overridden due to low BP or clinical symptoms. Even among the 11 treatments that saw five or more disregarded Controller suggestions, three treatments did not require a single override due to low BP or clinical symptoms, and more than half required two or fewer such overrides.

#### Direction and Magnitude of Deviations from Controller Suggestions

**Figure 5:**
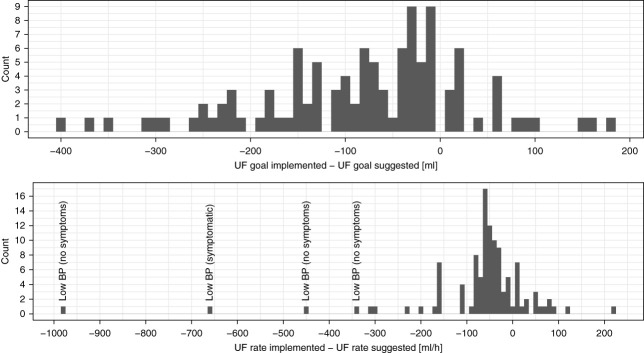
**Distribution of the differences between the Controller-suggested UF goals (top panel) and rates (bottom panel) and those implemented by the health care staff.** Underlying data: the 25 study visits (from 11 subjects) where the staff chose to disregard Controller suggestions. In the bottom panel, the reasons for disregarding the Controller suggestions are shown for the four largest differences between the suggested and implemented UF rates. UF, ultrafiltration.

Across the 56 study visits from 14 subjects where the Controller made UF suggestions (48 complete and eight incomplete visits), there were 26 visits (from 11 subjects) where at least one Controller recommendation was disregarded. In total, 111 Controller recommendations were disregarded, three of them in the form of UF pump stops. Figure 5 shows the magnitude and direction of the differences between implemented and suggested UF goals (top panel) and rates (bottom panel). For the top panel, the three UF pump stops were excluded (leaving 108 data points) because the UF goal on the machine was not changed, and a calculation of the difference between implemented and suggested UF goal is not meaningful in this context.

Of a total of 111 disregarded Controller suggestions, 20 implemented UF rates were *higher* than the respective Controller-suggested UF rates. Another 71 implemented UF rates were only <100 ml/h (on average 49 ml/h) lower than the respective Controller-suggested UF rates (*i.e*., rather mild differences). Together, these two categories made up 82% of all deviations between the Controller-suggested and the implemented UF rates, leaving only 20 more pronounced differences. Table 5 presents a breakdown of the reasons for the 111 Controller overrides that are depicted in Figure 5.

**Table 5:** Reasons the health care staff chose to disregard suggestions made by the Ultrafiltration Rate Feedback Controller

Reason for Disregarding the Controller Suggestion	No.
Health care staff preference (no clinical symptoms, no low BP)	65
Low BP (without clinical symptoms)	43
Low BP (with clinical symptoms)	3

Underlying data: all study visits with analyzable data where the Controller made ultrafiltration suggestions (56 visits).

## Discussion

**Figure 6:**
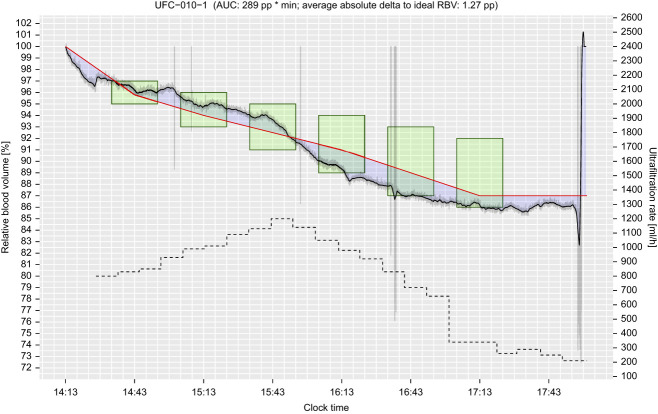
**Example study treatment (subject UFC-010, visit 1).** The solid black line represents the subject's filtered RBV curve (see Methods for details). Raw RBV values are shown in light gray. The red line denotes the desired RBV trajectory targeted by the Controller. The green boxes indicate the half-hourly RBV ranges associated with significantly improved patient survival, as published by Preciado *et al*.^8^ The dashed black line shows the UF rates suggested by the Controller. The plot title further provides the absolute AUC (pp×minutes) and average absolute difference between the filtered RBV curve and the desired RBV trajectory. AUC, area under the curve; pp, percentage points.

This study constitutes the first *in vivo* use of this adaptive UFR Feedback Controller prototype and demonstrates that this technology operated as intended. The Controller respected all limitations imposed on its operation, and all Skipped Updates, Fallback Conditions, and Hard Stops were triggered appropriately and handled correctly. It significantly increased the likelihood of RBV target range attainment compared with SOC treatments, with a clinically meaningful effect size. Figure 6 shows an example study treatment where the Controller can be seen modulating the UF rate throughout the dialysis session.

SBP and DBP (before, during, and after dialysis) did not differ significantly between study visits using the Controller and SOC treatments. However, the use of the Controller was associated with lower nadir systolic and DBP (systolic: 106 versus 111 mm Hg; diastolic: 55 versus 59 mm Hg). The clinical relevance of these differences remains uncertain. Given the variability in individual patient responses, we caution against overinterpretation of these findings and instead present them as part of a comprehensive characterization of the Controller's effects. Future studies with larger cohorts and clinical outcome measures will be needed to assess the true effect of these changes, particularly in the context of appropriately personalized Controller prescriptions (as would be used in clinical practice). Mechanistically, however, given that most patients have RBV slopes above the desired target range, these findings are not necessarily surprising, as the Controller will increase the UF goal in these patients to achieve a steeper RBV trajectory. Insufficient intradialytic fluid removal can reduce the likelihood of hypotensive episodes, albeit at the expense of worse long-term patient outcomes. Since the RBV trajectory targeted by the Controller was chosen for its association with lowest patient mortality, we hypothesize that a higher rate of attaining these RBV targets would lead to improved patient survival at the population level, even if associated with somewhat lower intradialytic BP. Larger prospective trials are needed to confirm this.

health care staff were instructed to only override Controller recommendations when medically necessary. However, this was not always followed. While the “health care staff preference” category certainly comprises situations where the staff, *e.g*., suspected impending hypotension and, therefore, proactively preferred lower UF rates, it also encompasses, by way of example, instances where the staff independently adjusted UF rates based on their clinical judgment to guide the RBV curve toward the target trajectory (and were, thus, essentially “competing” with the Controller), as well as instances where the staff did not implement Controller-based recommendations because the changes were deemed too minor to make a difference (*i.e*., where they had no objection to the recommendation but felt it was “not worth implementing”). The reasons for disregarding Controller recommendations were not captured in more detail beyond the categories listed in Table 5, preventing a quantitative analysis of medically versus nonmedically motivated overrides.

Overall, our study suggests that the Controller requires few enough interventions in most patients to make its use feasible in larger prospective trials. In most cases where the health care staff implemented UF rates that were different from the Controller's suggestion, the implemented UF rates were either only mildly lower or, in fact, *higher* than the Controller's suggestion. In addition, health care staff preference in the absence of low BP or clinical symptoms was the leading reason for not implementing Controller suggestions. It is not possible to know whether those interventions were necessary.

Despite the relatively small number of subjects, this study successfully demonstrated that this novel UFR Feedback Controller operated as expected in human subjects. Studying the effects of long-term use of this technology on target weight attainment, intradialytic complications, and patient survival was beyond the scope of this study.

In current practice, clinicians run treatments with a UF goal in a medically acceptable range and, typically, with a *constant* UF rate, irrespective of the patient's vascular refill kinetics. What the Controller adds is the individualization of UF based on the patient's evolving plasma refill characteristics during the treatment. It remains important, however, that the clinician define the medically appropriate UF goal range for the patient.

It would be desirable if a future iteration of the Controller could incorporate the patient's BP response during the treatment. Furthermore, bioelectrical impedance analysis, which is available in many countries around the world, although not yet in the United States,^11^ can not only inform clinical target weight decisions (and, thus, the Controller prescription to be used) but may also be incorporated into a future version the Controller itself in the form of individualized RBV target trajectories based on measured fluid overload. As a medium- to long-term goal, replacing RBV with *absolute* blood volume as the process variable for the Controller would almost certainly be superior. There are currently efforts underway to noninvasively determine absolute blood volume during hemodialysis,^12–18^ which, if successful, would pave the way for its use in this UFR Feedback Controller.

Commercially available feedback systems for hemodialysis include the Blood Volume Monitor by Fresenius Medical Care, the Hemocontrol biofeedback system by Gambro, and the Haemo-Master by Nikkiso.^19^ What distinguishes the novel feedback controller prototype evaluated in this study is its foundation on evidence-based RBV target ranges,^8,9^ setting it apart from existing systems.

The present pilot study confirms that this UFR Feedback Controller operates as intended and improves RBV target range attainment compared to current SOC, which lays the foundation for larger prospective trials to investigate the effect of this technology on patient survival and other clinically relevant outcomes.

## Supplementary Material

## Supplemental Material

Supplemental Table 1. RBV target ranges used for the analyses in this study.

Supplemental Table 2. Characteristics of study subjects with low versus high rate of disregarded Controller suggestions.

Supplemental Table 3. Information about incomplete study visits.
