# Supplementary material for: A Novel Ultrafiltration Rate Feedback Controller for Use in Hemodialysis: First Clinical Experience: An Interventional Pilot Study
Source: Kidney360. 2025 Jun 4;6(9):1562–72. doi: 10.34067/KID.0000000839 (PMC12483043; doi:10.34067/KID.0000000839)
Supplement: Supplementary file 1 [file kidney360-6-01562-s001.pdf]

ASN
American Society of Nephrology
JASN
Journal of the American Society of Nephrology
KIDNEY360
Accessing Our World From Every Angle
CJASN
Clinical Journal of the American Society of Nephrology

ASN Journal Disclosure Form

As per ASN journal policy, I have disclosed any financial relationships or commitments I have held in the past 36 months as included below. I have listed my Current Employer below to indicate there is a relationship requiring disclosure. If no relationship exists, my Current Employer is not listed.

S. Casper reports the following:
Employer: Fresenius Medical Care Deutschland GmbH; Research Funding: Fresenius Medical Care; and Patents or Royalties: Fresenius Medical Care.

I understand that the information above will be published within the journal article, if accepted, and that failure to comply and/or to accurately and completely report the potential financial conflicts of interest could lead to the following: 1) Prior to publication, article rejection, or 2) Post-publication, sanctions ranging from, but not limited to, issuing a correction, reporting the inaccurate information to the authors' institution, banning authors from submitting work to ASN journals for varying lengths of time, and/or retraction of the published work.

Name: Sabrina Casper
Manuscript ID: K360-2024-001060R1
Manuscript Title: A Novel Ultrafiltration Rate Feedback Controller for Use in Hemodialysis - First Clinical Experience (An Interventional Pilot Study)
Date of Completion: March 10, 2025
Disclosure Updated Date: March 10, 2025

ASN American Society of Nephrology | JASN Journal of the American Society of Nephrology | KIDNEY360 Accessing Our World From Every Angle | CJASN Clinical Journal of the American Society of Nephrology

ASN Journal Disclosure Form

As per ASN journal policy, I have disclosed any financial relationships or commitments I have held in the past 36 months as included below. I have listed my Current Employer below to indicate there is a relationship requiring disclosure. If no relationship exists, my Current Employer is not listed.

D. Fuertinger reports the following:
Employer: Fresenius Medical Care D-GmbH; Research Funding: Fresenius Medical Care; and Patents or Royalties: Multiple patents in the kidney space in the area of treatment of anemia, bone mineral disease, fluid management, and conduct of simulated clinical studies.

I understand that the information above will be published within the journal article, if accepted, and that failure to comply and/or to accurately and completely report the potential financial conflicts of interest could lead to the following: 1) Prior to publication, article rejection, or 2) Post-publication, sanctions ranging from, but not limited to, issuing a correction, reporting the inaccurate information to the authors' institution, banning authors from submitting work to ASN journals for varying lengths of time, and/or retraction of the published work.

Name: Doris H. Fuertinger
Manuscript ID: K360-2024-001060R1
Manuscript Title: A Novel Ultrafiltration Rate Feedback Controller for Use in Hemodialysis - First Clinical Experience (An Interventional Pilot Study
Date of Completion: March 10, 2025
Disclosure Updated Date: March 10, 2025

ASN American Society of Nephrology
JASN Journal of the American Society of Nephrology
KIDNEY360 Accessing Our World From Every Angle
CJASN Clinical Journal of the American Society of Nephrology

ASN Journal Disclosure Form

As per ASN journal policy, I have disclosed any financial relationships or commitments I have held in the past 36 months as included below. I have listed my Current Employer below to indicate there is a relationship requiring disclosure. If no relationship exists, my Current Employer is not listed.

S. Fuertinger reports the following:
Employer: Renal Research Institute (RRI) New York; Fresenius Medical Care; and Patents or Royalties: Fresenius Medical Care.

I understand that the information above will be published within the journal article, if accepted, and that failure to comply and/or to accurately and completely report the potential financial conflicts of interest could lead to the following: 1) Prior to publication, article rejection, or 2) Post-publication, sanctions ranging from, but not limited to, issuing a correction, reporting the inaccurate information to the authors' institution, banning authors from submitting work to ASN journals for varying lengths of time, and/or retraction of the published work.

Name: Stefan Fuertinger
Manuscript ID: K360-2024-001060R1
Manuscript Title: A Novel Ultrafiltration Rate Feedback Controller for Use in Hemodialysis - First Clinical Experience (An Interventional Pilot Study)
Date of Completion: March 11, 2025
Disclosure Updated Date: March 11, 2025

ASN American Society of Nephrology | JASN Journal of the American Society of Nephrology | KIDNEY360 Accessing Our World From Every Angle | CJASN Clinical Journal of the American Society of Nephrology

ASN Journal Disclosure Form

As per ASN journal policy, I have disclosed any financial relationships or commitments I have held in the past 36 months as included below. I have listed my Current Employer below to indicate there is a relationship requiring disclosure. If no relationship exists, my Current Employer is not listed.

P. Kotanko reports the following:
Employer: Renal Research Institute; Ownership Interest: Fresenius Medical Care; Research Funding: Fresenius Medical Care; NIH; KidneyX; PCORI; Patents or Royalties: Multiple patents in the kidney space; and Advisory or Leadership Role: Editorial Board of Blood Purification; Editorial Board of Kidney and Blood Pressure Research; Editorial Board of Frontiers in Nephrology.

I understand that the information above will be published within the journal article, if accepted, and that failure to comply and/or to accurately and completely report the potential financial conflicts of interest could lead to the following: 1) Prior to publication, article rejection, or 2) Post-publication, sanctions ranging from, but not limited to, issuing a correction, reporting the inaccurate information to the authors' institution, banning authors from submitting work to ASN journals for varying lengths of time, and/or retraction of the published work.

Name: Peter Kotanko
Manuscript ID: K360-2024-001060R1
Manuscript Title: A Novel Ultrafiltration Rate Feedback Controller for Use in Hemodialysis - First Clinical Experience
Date of Completion: April 17, 2025
Disclosure Updated Date: April 17, 2025

ASN American Society of Nephrology
JASN Journal of the American Society of Nephrology
KIDNEY360 Accessing Our World From Every Angle
CJASN Clinical Journal of the American Society of Nephrology

ASN Journal Disclosure Form

As per ASN journal policy, I have disclosed any financial relationships or commitments I have held in the past 36 months as included below. I have listed my Current Employer below to indicate there is a relationship requiring disclosure. If no relationship exists, my Current Employer is not listed.

L. Rivera Fuentes reports the following:
Employer: Renal Research Institute, LLC; Applied Therapeutics, Inc; Omeros Corporation; Ownership Interest: Applied Therapeutics, Inc; Omeros Corporation; Research Funding: Renal Research Institute, LLC; Applied Therapeutics, Inc; Omeros Corporation; and Honoraria: Renal Research Institute, LLC; Applied Therapeutics, Inc; Omeros Corporation.

I understand that the information above will be published within the journal article, if accepted, and that failure to comply and/or to accurately and completely report the potential financial conflicts of interest could lead to the following: 1) Prior to publication, article rejection, or 2) Post-publication, sanctions ranging from, but not limited to, issuing a correction, reporting the inaccurate information to the authors' institution, banning authors from submitting work to ASN journals for varying lengths of time, and/or retraction of the published work.

Name: Lemuel Rivera Fuentes
Manuscript ID: K360-2024-001060R1
Manuscript Title: A Novel Ultrafiltration Rate Feedback Controller for Use in Hemodialysis - First Clinical Experience (An Interventional Pilot Study)
Date of Completion: March 12, 2025
Disclosure Updated Date: March 12, 2025

ASN American Society of Nephrology
JASN Journal of the American Society of Nephrology
KIDNEY360 Accessing Our World From Every Angle
CJASN Clinical Journal of the American Society of Nephrology

ASN Journal Disclosure Form

As per ASN journal policy, I have disclosed any financial relationships or commitments I have held in the past 36 months as included below. I have listed my Current Employer below to indicate there is a relationship requiring disclosure. If no relationship exists, my Current Employer is not listed.

L. Tapia Silva reports the following:
Employer: Astra Zeneca AB R&D; and Patents or Royalties: Fresenius Medical Care.

I understand that the information above will be published within the journal article, if accepted, and that failure to comply and/or to accurately and completely report the potential financial conflicts of interest could lead to the following: 1) Prior to publication, article rejection, or 2) Post-publication, sanctions ranging from, but not limited to, issuing a correction, reporting the inaccurate information to the authors' institution, banning authors from submitting work to ASN journals for varying lengths of time, and/or retraction of the published work.

Name: Leticia Mirell Tapia Silva
Manuscript ID: K360-2024-001060R1
Manuscript Title: A Novel Ultrafiltration Rate Feedback Controller for Use in Hemodialysis - First Clinical Experience (An Interventional Pilot Study)
Date of Completion: March 15, 2025
Disclosure Updated Date: March 15, 2025

ASN American Society of Nephrology | JASN Journal of the American Society of Nephrology | KIDNEY360 Accessing Our World From Every Angle | CJASN Clinical Journal of the American Society of Nephrology

ASN Journal Disclosure Form

As per ASN journal policy, I have disclosed any financial relationships or commitments I have held in the past 36 months as included below. I have listed my Current Employer below to indicate there is a relationship requiring disclosure. If no relationship exists, my Current Employer is not listed.

S. Thijssen reports the following:
Employer: Renal Research Institute (affiliated with Fresenius Medical Care North America); Research Funding: Fresenius Medical Care; Patents or Royalties: Fresenius Medical Care Holdings, Inc.; Fresenius Medical Care has patents and patent applications in various stages of the patent prosecution process. I am an inventor on some of these patents and/or patent applications but have assigned my ownership interest to Fresenius Medical Care. I have received and/or may be entitled to a nominal patent award per Fresenius Medical Care North America policy, but I receive no royalties and have no ownership interest in any of the intellectual property.; and Other Interests or Relationships: I hold performance shares (virtual shares) in Fresenius Medical Care.

I understand that the information above will be published within the journal article, if accepted, and that failure to comply and/or to accurately and completely report the potential financial conflicts of interest could lead to the following: 1) Prior to publication, article rejection, or 2) Post-publication, sanctions ranging from, but not limited to, issuing a correction, reporting the inaccurate information to the authors' institution, banning authors from submitting work to ASN journals for varying lengths of time, and/or retraction of the published work.

Name: Stephan Thijssen
Manuscript ID: K360-2024-001060R1
Manuscript Title: A Novel Ultrafiltration Rate Feedback Controller for Use in Hemodialysis - First Clinical Experience (An Interventional Pilot Study)
Date of Completion: March 27, 2025
Disclosure Updated Date: March 27, 2025

ASN American Society of Nephrology
JASN Journal of the American Society of Nephrology
KIDNEY360 Accessing Our World From Every Angle
CJASN Clinical Journal of the American Society of Nephrology

ASN Journal Disclosure Form

As per ASN journal policy, I have disclosed any financial relationships or commitments I have held in the past 36 months as included below. I have listed my Current Employer below to indicate there is a relationship requiring disclosure. If no relationship exists, my Current Employer is not listed.

X. Ye reports the following:
Employer: Renal Research Institute

I understand that the information above will be published within the journal article, if accepted, and that failure to comply and/or to accurately and completely report the potential financial conflicts of interest could lead to the following: 1) Prior to publication, article rejection, or 2) Post-publication, sanctions ranging from, but not limited to, issuing a correction, reporting the inaccurate information to the authors' institution, banning authors from submitting work to ASN journals for varying lengths of time, and/or retraction of the published work.

Name: Xiaoling Ye
Manuscript ID: K360-2024-001060R1
Manuscript Title: A Novel Ultrafiltration Rate Feedback Controller for Use in Hemodialysis - First Clinical Experience (An Interventional Pilot Study)
Date of Completion: March 15, 2025
Disclosure Updated Date: March 15, 2025
