# Supplementary material for: A Novel Ultrafiltration Rate Feedback Controller for Use in Hemodialysis: First Clinical Experience: An Interventional Pilot Study
Source: Kidney360. 2025 Jun 4;6(9):1562–72. doi: 10.34067/KID.0000000839 (PMC12483043; doi:10.34067/KID.0000000839)
Supplement: Supplementary file 2 [file kidney360-6-01562-s002.pdf]

Supplemental Material

Table of Contents for Supplemental Material

- **Supplemental Table 1: Relative blood volume target ranges used for the analyses in this study**
- **Supplemental Table 2: Characteristics of study subjects with low vs. high rate of disregarded controller suggestions**
- **Supplemental Table 3: Information about incomplete study visits**

Supplemental Tables

Timepoint	RBV target range [%]	RBV with lowest all-cause mortality [%]
30	95 to 97	95.5
60	93 to 96	94
90	91 to 95	92.5
120	89 to 94	91
150	87 to 93	89
180	86 to 92	87

Supplemental Table 1: Relative blood volume target ranges used for the analyses in this study

In a study by Preciado *et al.*[8], patients with relative blood volume (RBV) curves within these ranges were found to have significantly lower all-cause mortality compared to patients outside of these ranges. The RBV values associated with the lowest all-cause mortality are rounded to the nearest 0.5%.

Parameter	Subjects with low rate of disregarded Controller suggestions	Subjects with high rate of disregarded Controller suggestions	P value
Number of subjects	9	6	n/a
Age [years]	59 ± 16	58 ± 16	0.92
Sex: female [%]	44	50	1
Dialysis vintage [years]	3 ± 1.6 (min 0.5; max 4.8)	5.7 ± 2.7 (min 2.3; max 9.9)	0.063
Body Mass Index [kg/m^2]	30.9 ± 14.9 (min 18.5; max 67.3)	29.7 ± 7.4 (min 20.8; max 43.4)	0.69
Baseline IDWG [kg]	2.7 ± 0.7	2.4 ± 0.9	0.43
Baseline Pre-HD Weight [kg]	88.4 ± 42.1	85.3 ± 25.9	1
Baseline Post-HD Weight [kg]	85.6 ± 41.6	82.6 ± 25.4	0.95
Baseline Clin. Target Weight [kg]	84.4 ± 41.6	81.7 ± 25.7	1
Prescribed Treatment Time [min]	228 ± 28	213 ± 29	0.58

Supplemental Table 2: Characteristics of Study Subjects With Low vs. High Rate of Disregarded Controller Suggestions. ("High rate" subjects had study treatments with > 30% disregarded Controller suggestions; "low rate" group are all other subjects.)

Subjects in the "high rate" group were defined as those who contributed study treatments during which more than 30% of Controller suggestions were disregarded (those were seven study visits, corresponding to the seven right-most bars in Figure 4, contributed by six unique subjects); subjects in the "low rate" group are all other subjects. The association between sex (female/male) and disregarded Controller suggestion group (high/low) was assessed with Fisher's exact test. Based on normality testing, age, vintage, and interdialytic weight gain were compared using Welch's test, and all other parameters were compared using Mann-Whitney U test. Baseline parameters were defined as the average of the three values from the most recent dialysis sessions after the long, first short, and second short interdialytic interval before the first study visit. Body mass index was calculated using the baseline post-dialysis weight. HD, hemodialysis; IDWG, interdialytic weight gain.

Study visit ID	Controller stop time [min]	Controller stop type	Reason for Controller stop
UFC-008-4	32.8	Fallback	Access recirculation test led to UFR of 70 ml/min and triggered Fallback.
UFC-013-2	13.4	Hard Stop	Crit-Line® cable not properly plugged in. Controller restart required. At that point, remaining UF time was below the allowed minimum.
UFC-007-8	13.4	Fallback	Crit-Line® data not stable. Controller properly entered Fallback mode.
UFC-009-3	51.0	Hard Stop	Healthcare staff manually increased the UF goal beyond the allowed limit.
UFC-005-1	13.8	Hard Stop	Prescribed UF time greater than allowed.
UFC-001-1	81.4	Hard Stop	Problem reading 2008T machine data file.
UFC-001-2	83.3	Hard Stop	Problem reading 2008T machine data file.
UFC-010-5	32.8	Hard Stop	Problem reading 2008T machine data file.
UFC-011-3	23.5	Hard Stop	Problem reading 2008T machine data file.
UFC-014-1	23.2	Hard Stop	Problem reading 2008T machine data file.
UFC-010-3	176.0	Fallback	Remaining UF time was increased beyond the allowed limit.

Supplemental Table 3: Information about incomplete study visits

In only one case did a medical intervention lead to the automatic disengagement of the Controller (UFC-010-3: UF pump was stopped for approximately 40 minutes due to low blood pressure; when the UF pump was restarted, the remaining UF time exceeded the allowed value for use of the Controller). UF, ultrafiltration; UFR, ultrafiltration rate.
